# Baló's concentric sclerosis in a girl with interesting presentation

**Published:** 2013

**Authors:** Leila Aghaghazvini, Anahita Sadeghi, Bahman Rasuli, Shirin Aghaghazvini

**Affiliations:** 1 Assistant Professor, Department of Radiology, School of Medicine AND Shariati Hospital, Tehran University of Medical Sciences, Tehran, Iran; 2 Assistant Professor, Department of Internal Medicine, School of Medicine AND Shariati Hospital, Tehran University of Medical Sciences, Tehran, Iran; 3 Resident, Department of Radiology, School of Medicine, Tehran University of Medical Sciences, Tehran, Iran; 4 General Physician, Advanced Diagnostic and Interventional Research Center, Tehran University of Medical Sciences, Tehran, Iran

**Keywords:** Balo's Concentric Sclerosis, Multiple Sclerosis, Brain, MRI, Low Grade Glioma, Abscess

## Abstract

Balo's concentric multiple scleroses (MS) is a rare demyelinating disease and a variant of multiple sclerosis. We report a case with interesting misleading clinical history and typical RI findings of Balo disease. A 19-year-old girl presented with fever and left hemiparesis following dental procedure 15 days ago. On physical examination fever and left limbs forces loss were noted. On CT scan a hypodense mass like area, and in MRI a low T1 and high T2, and flair concentric onion-like partial enhancement of mass in parieto-frontal periventricular white matter was detected. Regarding the findings tumefactive MS, Low grade glioma, lymphoma, and abscess and regarding the history of abscess formation were in differential diagnosis. After therapy and no improvement in clinical condition and MRI findings during 2 months, the patient underwent stereotactic biopsy and tumefactive balo MS was revealed. After 4 months of intensive therapy, the patient was discharged with normal condition.

## Introduction

Balo's concentric sclerosis (BCS) is a tumefactive demyelinating lesion of the central nervous system (CNS) and considered a rare variant of multiple sclerosis (MS).

The clinical course is characterized by an acute onset and steady progression to major disability within a few months. Age of onset is between 20–50 years, and patients predominantly present with cerebral symptoms. Classical manifestations of the disease are headache, aphasia, cognitive or behavioral dysfunction, and/or seizures. Most patients are young adults in their second to fifth decades of life, and it is relatively more common in males.^1^

BCS is delineated pathologically by huge, tumor-like brain lesions showing concentric rings of alternating demyelination and preserved myelin layers which involve the cerebral hemispheres, cerebellum, brain stem, spinal cord, and optic chiasm.^1, 2^

Magnetic resonance imaging (MRI) associated with BCS demonstrates concentric rings on T2-weighted and contrast-enhanced T1-weighted images, and thus helps differentiation from other pathology. T2-weighted MRI scans sometimes reveal a distinct pattern of hypo-/isointense and hyperintense rings corresponding to bands of preserved and destroyed myelin.^1–3^ Diagnosis is based on clinical signs and symptoms consistent with BCS, exclusion of other neurologic diseases, and the characteristic concentric rings on MR images. In the present case report, we report the case of a 19-year-old female with BCS involving right parieto-frontal periventricular white matter, with typical MRI appearance but interesting misleading clinical history.

## Case Report

**Figure 1:**
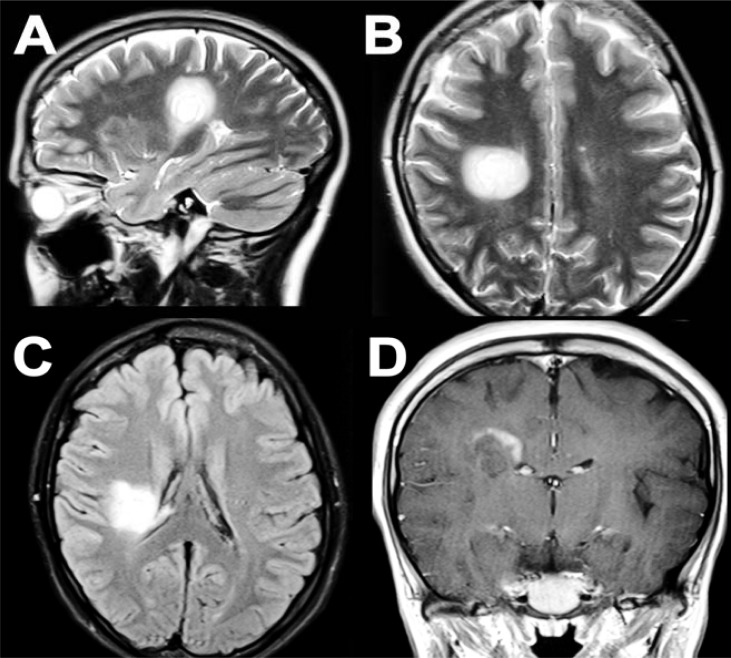
Sagittal and axial T2-weighted brain MRI showing hyperintense concentric onion-like mass in the right parieto-frontal periventricular white matter (A,B), axial FLAIR MRI showing hyperintense concentric pattern in the right parieto-frontal periventricular white matter (C), and coronal post-contrast T1-weighted image demonstrating some open ring enhancement of the lesion in the right parieto-frontal area

A 19-year-old girl referred with a history of decrease in left limbs forces, muscular tones, and fever following dental procedure from 15 days ago. Urgent plain computed tomography of brain revealed a hypodense mass-like area in the right parieto-frontal periventricular white matter. Brain MRI revealed a tumefactive lesion in the right parieto-frontal periventricular white matter with alternating hypo-isointense and hyperintense concentric onion-like bands on T1- and T2-weighted and FLAIR, and partial enhancement in post-contrast images in parieto-frontal periventricular white matter with mild edema and no hemorrhage and restriction (Figure 1).

According to history and imaging findings tumefactive MS, Low grade glioma, and lymphoma was our differential diagnosis; however, only regarding the patient's history, abscess formation was the most possible diagnosis. The patient received intravenous antibiotic and modified steroid therapy. No improvement was observed in clinical conditions or MRI findings during 2 months. The patient underwent stereotactic biopsy to differentiate probable tumoral lesion from abscess or tumefactive MS. Biopsy revealed Tumefactive Balo MS. After 4 months of intensive therapy, the patient was discharged in a good condition. Our patient did not experience any relapse, and at the time of the last follow-up examination a year after starting treatment, she had only residual mild left hemiparesis.

## Discussion

BCS is considered as a rare variant of inflammatory demyelinating disorder that presents the characteristic concentric area of alternating demyelination and myelination on pathologic examination with an apparent basic pathologic similarity to multiple sclerosis. However, the exception of an alternating lamellar pattern of demyelinated and well-preserved white matter which encompass alternating bands of low and high signal in white matter were seen on T2-weighted images. The presence of concentric ring enhancement was also observed on post contrast T1-weighted images. Edema and mass effect may also be seen.^1–4^ In our case, concentric pattern on all MR sequences and partial enhancement of mass lesion on post-gadolinium scan were seen. The exact reason for BCS lesion formation is not clear. Recently, it has been proposed that demyelination in these lesions are similar to hypoxic-like tissue injury.

Clinical differential diagnosis includes acute disseminated encephalomyelitis (ADEM), multiple sclerosis (MS), neoplasms, and infections such as abscesses. However, the typical concentric pattern on MR images is highly suggestive of BCS. The concentric pattern is not always observed if the MR imaging is not performed early in the course of the disease.^1, 2^

A brain biopsy should then be considered to exclude other disorders. In histopathological investigation, it is important to stain for myelin to determine alternating concentric zones of demyelination.^4^

In our patient, in regard to history, Imaging findings, and the clinical process following primary therapy (no response in our case), tumefactive MS, neoplasia lesion, and infection were in differential diagnosis.

Corticosteroids at high dosage are the first line of therapy and have been shown to be effective in recovery from neurological deficits related to BCS. Our patient had a good response to intensive steroid therapy during the 4 months after starting of treatment. Often, steroids do not alter the acute attacks of CNS demyelination, but cycles of plasmapheresis have been reported to be useful. Some cases have been treated successfully with immunosuppressive drugs, such as mitoxantrone.^2^ In conclusion, BCS disease can mimic space-occupying lesions in the central nervous system with similar clinical and radiological features.
